# Implementation of remote general movement assessment using the in-motion instructions in a high-risk norwegian cohort

**DOI:** 10.1186/s12887-024-04927-4

**Published:** 2024-07-10

**Authors:** Lars Adde, Kristin Bjørnstad Åberg, Toril Fjørtoft, Kristine Hermansen Grunewaldt, Randi Lade, Siril Osland, Frank Piegsa, Per Gunnar Sandstrøm, Ragnhild Støen, Gunfrid V. Størvold, Beate Horsberg Eriksen

**Affiliations:** 1 Department of Clinical and Molecular Medicine, Norwegian University of Science and Technology, Trondheim, Norway; 2 Clinic of Rehabilitation, St. Olavs Hospital, Trondheim University Hospital, Trondheim, Norway; 3 Department of Pediatrics, Møre and Romsdal Hospital Trust, Ålesund Hospital, Ålesund, Norway; 4 Nord Trondelag Hospital Trust, Habilitation Centre, Levanger, Norway; 5 Department of Neonatology, St. Olavs Hospital, Trondheim University Hospital, Trondheim, Norway; 6 Department of Public Health and Nursing, Norwegian University of Science and Technology, Trondheim, Norway

**Keywords:** Cerebral palsy, Early detection, High-risk infant, General movement assessment

## Abstract

**Background:**

General Movement Assessment (GMA) is recommended for early detection of risk for cerebral palsy but requires trained clinical experts. We aimed to implement home- and hospital-based filming for remote GMA in a Norwegian high-risk infant cohort, as well as evaluating parents’ experiences in filming their infant at home.

**Methods:**

This knowledge translational study used a prospective cohort design including participants referred to neurodevelopmental follow-up across three sites in the Central Norway Regional Health Authority. Two home films of the fidgety type of general movements were collected between 12^+1^–14^+6^ and 15^+1^–17^+6^ weeks after term by parents. An additional film was collected at the hospital between 12^+1^ and 17^+6^ weeks after term. The instructional guide for all filming was the In-Motion App standards. Videos were transferred to a remote GMA team and classified as either “GMA scorable” or “GMA not scorable” based on Prechtl’s GMA standards. Parents responded to an online survey using a 5-point Likert scale to collect information about their perspectives, experiences, and possible worries by filming their infant at home.

**Results:**

One-hundred-and-two infants from 95 families participated. Ninety-two (96.8%) families transferred 177 home-based videos. Eighty-four (92%) of these had 95 videos taken in their local hospital. All 177 home-videos were “GMA scorable” and three (3,1%) out of 95 hospital-based videos were classified as “GMA not scorable”. Eight families did not respond to the survey and two families did not receive the survey due to a technical error. Seventy-eight (91.7%) respondents agreed or strongly agreed that it was easy to perform home filming and five (5.9%) agreed that they were more worried about their child`s development after filming at home. Almost 80% of respondents agreed that a video for GMA can be taken at home instead of in hospital.

**Conclusions:**

This study strengthens the clinical implementation of home filming by parents and remote GMA for early detection of CP in high-risk follow-up programs. The implementation of remote GMA has the potential to facilitate early intervention to improve function in children with CP in line with international recommendations.

**Trial registration:**

ClinicalTrials.gov ID: NCT04287166 Date of registration: 27/02/2020.

**Supplementary Information:**

The online version contains supplementary material available at 10.1186/s12887-024-04927-4.

## Background

Cerebral palsy (CP) is the most common childhood disability, causing functional limitations and co-occurring impairments due to injury to the developing brain [1, 2]. Diagnosis is typically made at 12 – 24 months of age in high-income countries and as late as 5 years of age in low- and middle-income countries [3–5]. Follow-up in high-risk infants has the goal to provide early identification and referral for targeted interventions to infants with CP [6].

Using standardized follow-up to identify infants with increased risk of CP based on their clinical history gives opportunity to provide interventions during infancy when neuroplasticity is high. Such early identification will improve access to community health services for those who may benefit and reassure parents of those infants who are unlikely to develop CP [7–10]. An early “high-risk of CP” warning before 5 months of age by combining results from neonatal neuroimaging, the Prechtl General Movement Assessment (GMA) and the Hammersmith Infant Neurological Examination (HINE), can decrease the age of CP diagnosis [5, 11]. A cornerstone in early detection of CP is the GMA due to the fact that it is most predictive for CP, non-invasive and cost-efficient [12]. The GMA is based on visual observation of whether the fidgety type (FMs) of General Movements (GMs) are present or not between 12 to 18 weeks after term age in 3–5-min-long video recordings [12, 13]. The GMA has shown high accuracy in detection of CP before 5 months after term and is today recommended for clinical use [5, 14–17].

Neurodevelopmental follow-up after discharge from the neonatal intensive care unit (NICU) is usually designed as visits to the out-patient clinic at the hospital [18]. Consequently, filming for infant FMs between 12 and 18 weeks after term are typically done by trained clinicians within face-to-face clinical settings [19]. Driven by the need to empower parents, reduce the need for clinical hospital appointments (augmented by both the COVID-19 pandemic and the need to reduce costs), overcome geographical travel distance, and improve the quality of video recordings by letting families choose the best timing and surroundings for filming their infant, new telehealth solutions have recently been developed [20, 21].

Smartphone apps like the Baby Moves, In-Motion and NeuroMotion, have been used successfully by parents for home-based filming of GMs [22–25]. The app solutions enable remote GMA by trained experts located at specialized hospital centers. However, the use of smartphone apps have been limited to research settings and app production and operating expenses are costly [19]. In addition, the possibility to share GMA knowledge and films between hospitals regardless of GMA expertise, and in cases where caregivers cannot film their baby at home, is often hampered by lack of legislation consensus on data storage and transfer policy. Data transfer policy often differs between research and clinical settings, and storage and transmission of digital images like GMs films need to comply to local legislation and clinical organizational policies [19]. Equally important is the need for evaluation of parental experiences and perspectives in filming their infant’s spontaneous movements at home for remote CP risk assessment.

The aim of this study was to implement home- and local hospital-based filming of GMs for remote expert-based GMA in follow-up programs in the Central Norway Regional Health Authority. We also wanted to evaluate the proportion of hospital films and family films performed in a Norwegian high-risk infant cohort that complied with standards for GMA, as well as parents’ experiences in filming their infant at home. We hypothesized that more than 80% of families returned home videos that correctly complied with GMA standards and that parents were not more stressed performing home videos.

## Methods

This knowledge translational study used a prospective cohort design and included a knowledge translation action plan developed in cooperation with user and health-care representatives [20, 26]. The study was developed based on five questions presented by Lavis et. al. [27] for the development of knowledge translation actions. An additional file shows key questions and responses (see Additional file 1). The plan was developed by addressing the needs of each hospital organization and their follow-up health-care teams. The chief investigator and investigator team performed a barrier analysis by interviewing key members of the follow-up health-care teams at the three participating hospitals. One out of three participating hospitals had GMA trained personnel available for filming. Several barriers to the implementation of hospital- and home filming and remote GMA were identified, including lack of clinical time for the remote GMA team, lack of digital apps for smartphone hospital filming, and lack of a digital healthcare solution for transfer of home-based films that complied with legislation consensus on data storage and video transfer policy. The identified barriers were met with a knowledge translation action plan to stimulate clinical uptake of the solution. An additional document shows this in more details (see Additional file 2).

### Participants

Participants were recruited from NICU`s across three sites (St. Olavs Hospital, Ålesund Hospital and Levanger Hospital) in the Central Norway Regional Health Authority. We aimed to recruit 100 infants. Infants were included if they were admitted to one of the three participating NICUs and referred to neurodevelopmental follow-up at discharge from 1st of August 2020 to 1st of August 2022. The centers had slightly different criteria for follow-up, but all places referred neonates considered at moderate-to-high risk of adverse neurodevelopmental development (prematurity (birth weight (BW) < 1500 g and/or gestational age (GA) < 32 weeks (one hospital) or BW < 1000 g and/or GA < 28 weeks (two hospitals), neonatal arterial ischemic stroke, hypoxic-ischemic encephalopathy (HIE) with- and without therapeutic hypothermia or other neonatal conditions affecting the risk for perinatal brain injury).

### Data collection procedure

Infant demographic data and perinatal medical data were collected from medical records and stored in a web-based data retrieval solution at St. Olavs University Hospital. Two separate home films taken between 12^+1^–14^+6^ and 15^+1^–17^+6^ weeks after term were collected to ensure that GM videos were taken during the period for the fidgety type of GMs. After receiving home films, parents were contacted with a link to an online survey to collect information about their perspectives, experiences and possible worries due to filming their infant at home. To facilitate implementation of hospital filming if parents could not film at home, a parallel film between 12^+1^ and 17^+6^ weeks after term was taken by a physiotherapist/nurse during the scheduled appointment in the outpatient follow-up hospital program. The instruction for healthcare personnel was to record the first video between week 12^+1^ and 14^+6^ after term and a second video between week 15^+1^ and 17^+6^ after term if abnormal GMA was identified on the first video. The hospital-based film was also transferred to the remote GMA team for assessment.

### Information to parents/caregivers

Parents were contacted by a local physiotherapist/nurse/pediatrician before discharge from the NICU. Information was given regarding home filming procedures, the use of remote GMA for early risk for CP assessment, that they would receive phone calls from the remote GMA team with more detailed information, and that they would be scheduled for an appointment with their local follow-up team when the infant was between 12 to 18 weeks after term. When infants were about 10 weeks after term, the first follow-up phone from the remote GMA team was performed giving more detailed information about the home filming procedures. The use of a digital healthcare solution providing SMS-messages was explained and parents were informed that all results from the GMA risk assessment would be communicated to them by the local follow-up team as part of the routine clinical appointment they would have at the hospital. A second follow-up phone call to the parents by the remote GMA team was performed by the end of week 14 after term or the beginning of week 15 after term to pick up any questions about the home filming procedures.

### Digital health care solution, hospital smartphone app and instructional guidelines

For home-based recordings, a commercially available digital health care solution (CheckWare), already approved for transfer of patient data in the Central Norway Regional Health Authority, was used. The solution was customized with a digital “treatment plan” for the communication with parents and transfer of home-based videos to the remote GMA team at St. Olavs Hospital. Automated SMSs were generated to remind parents to film their infant and digital alerts on a desk-top application was provided to the remote GMA team whenever a film was uploaded. The SMS informed parents about where to find the video-based instructional guides on YouTube. Reminders to parents were provided every 7th day if a video was not uploaded to the hospital server.

For hospital-based recordings, a solution with an existing hospital video capture app (VidiView) was implemented. The follow-up teams at each of the three participating hospitals received training in using the video capture app which was installed on hospital smart phones. The video was automatically transferred from the smart phone to the remote GMA-team’s hospital server.

Healthcare personnel from two of the three participating hospitals and all parents had access to the same instructional video for GM filming through a Uniform Resource Locator (URL) link. Instructions for recording a video of 3 min were the same for both parents and healthcare personnel. The instructional guideline video had been developed with consumer involvement by the Norwegian Cerebral Palsy organization and was the same as previously published and feasibility tested in the In-Motion App study [23]. Parents and healthcare personnel could observe the instructional video for information about standards for GMA (infant state, light, clothing, positioning, and duration of video) as many times as needed. The hospital with the remote GMA team had access to trained and experienced GMA personnel who did not need the instructional video.

### Assessment of video quality and GMs

All videos were assessed by a certified GMA observer in the remote GMA team with respect to the following standards [13]: 1) active movements (not hypokinetic), supine position, correct state, adequate clothing (diaper or a onesie), no disturbances during recording, sufficient light, whole body visible in video frame and correct position of smartphone camera. Videos were classified as either “GMA scorable” or “GMA not scorable” based on these standards [23].

The remote GMA team had three certified and experienced GMA observers who met once a week to observe incoming videos. None of them had knowledge about the infants clinical history if infant video was from St. Olavs hospital and at least two had no knowledge about the clinical history if infant video was from St. Olavs hospital. The three GMA observers observed all videos independently, and in cases of disagreement, a majority decision was made and used as the final classification. Fidgety type of general movements was assessed according to Prechtl GMA [13]. Infants’ movements were classified as normal if fidgety movements (FMs) were present (continuous (FM + +) or intermittent (FM +)). Infants were classified as having abnormal movements if FMs were sporadic (FM ±), abnormal (Fa) or absent (FM-) [12, 13].

The final GMA classification was documented in the child’s medical record by a member of the remote GMA team (medical records for all children included in the study were made available to the remote GMA team for documentation). The local follow-up team was responsible for looking up the results documented in the child’s medical record. However, if the GMA result was abnormal indicating a high risk for CP, this was communicated to the local pediatrician by the remote GMA team by a phone call. Further evaluation of risk for CP and follow-up including the GMA and other assessment result was managed by the local hospital team and evaluation of possible clinical consequences for the individual child was not a part of this study.

### Parental survey

A digital survey was developed to assess parents’ perspectives, experiences and possible worries related to filming their infant at home for remote CP risk assessment. Only one parent in the family could reply to the survey. The survey was designed with different statements, and parents indicated agreement or disagreement with the statement on a 5-point Likert scale. An additional survey file shows this in more detail (see Additional file 3). The digital survey solution provided security measures to ensure data accuracy and privacy.

### Statistical analysis

Data were analyzed using SPSS statistics V.29.0 (IBM SPSS Statistics). Data are presented as numbers with percentages (%) or with mean with SD and range. Descriptive statistics summarizes participants characteristics and survey responses. GMA results will be presented with numbers and proportions (%).

## Results

**Fig. 1:**
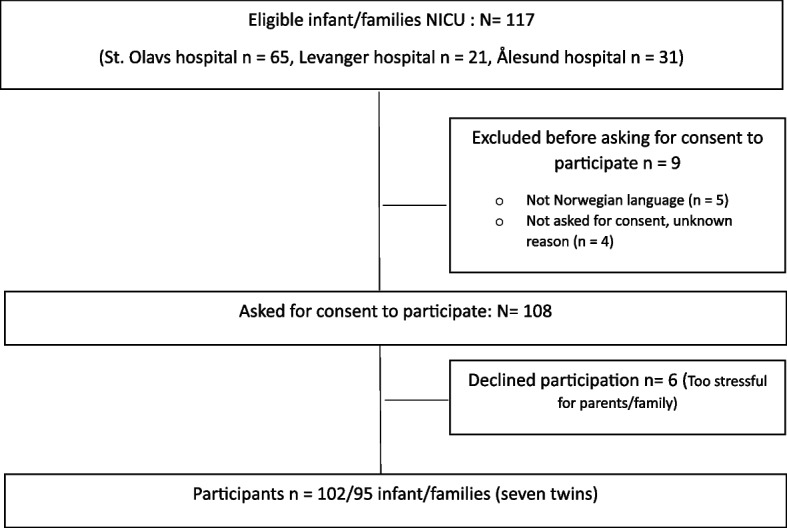
Flow-chart of participants

The recruitment and flow of participants are described in Fig. 1. In total, 102 infants from 95 families (seven twins) were recruited. Infant and family characteristics are shown in Table 1.

**Table 1:** Infant/family characteristics

	Infants (*n* = 102)
**Birth characteristics**	
Gestational age, weeks, mean (SD, range)	30.6 (5.4, 23–41)
Birthweight, g, mean (SD, range)	1778 (1276, 445–5920)
Male gender, n (%)	55 (54%)
**Perinatal data**	
Neonatal ischemic stroke, n (%)	5 (4.9)
Perinatal asphyxia without therapeutic hypothermia, n (%)	5 (4.9)
Perinatal asphyxia with therapeutic hypothermia, n (%)	7 (6.9)
Gestational age < 28 weeks, n (%)	37 (36.3)
Gestational age 28–31 weeks, n (%)	36 (35.3)
Gestational age 32–41 weeks, n (%)	29 (28.4)
	Families (*n* = 85)*
**Socio-demographic data,** ***n*** **(%)**	
Mother responding to survey, *n* (%)	72 (84.7)
Married/cohabiting family, *n* (%)	83 (97.6)
Mean age years survey respondent (SD, range)	31.8 (6.2/, 21–56)
Mean age years survey partner (SD, range)	32.6 (5.5, 21–48)
Single child, *n* (%)	44 (51.7)
**iOS vs. Android**	
iOS users, *n* (%)	51(60)

### Challenges to knowledge translation

Most of the challenges were related to technical issues in the knowledge translation action plan. Occasionally, technical procedures updating the smartphone app (VidiView) used for hospital-based filming introduced app malfunction and made filming of the infant impossible. In these cases, a back-up smartphone was used. Also, there was an occasional problem with transfer of video recordings from parents’ smartphones to the hospital server if the video file exceeded a pre-set file size limit of 500 megabytes. In such cases, someone from the remote GMA team called the parents and asked them to save the video with a lower size limit and transfer the video file again.. Organization of weekly expert-based GMA meetings, phone call procedures with parents, SMS communication between hospital and parents and accessibility to the In-Motion instructional video progressed without noticeable negative events.

### Video recordings

**Fig. 2:**
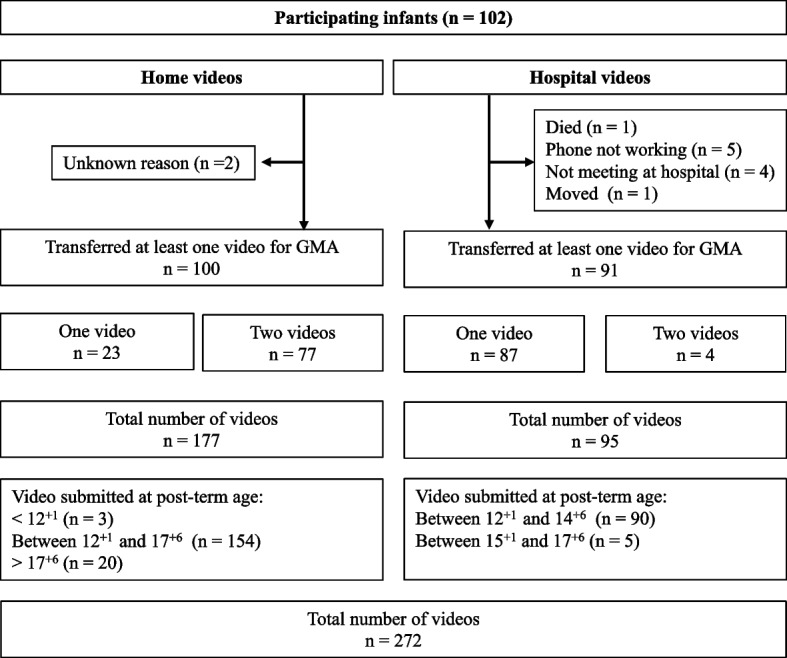
Flow chart of return rate of videos

Ninety-two (96.8%) of 95 included families transferred a total of 177 home-based videos. In parallel, 84 (92%) of these had a total of 95 videos taken in their local hospital and transferred to the remote GMA team. Two hundred and eleven (77.5%) of the 272 videos taken by both parents and healthcare personnel were within the targeted length of 3 min ± 5 s. Six (2.2%) videos were shorter than this, and 12 (4.4%) videos were longer than 4 min. The video return rate where at least one video was returned was 100 (98%) in videos taken at home and 91 (89.2%) in videos taken at hospital. Ninety (94.7%) of 95 infant hospital videos were transferred correctly by healthcare personnel within first time window between week 12^+1^ and 14^+6^ after term. Details of return rate of videos and reasons for non-transfer of videos are shown in detail in Fig. 2.

### Video quality for remote GMA

All 177 uploaded home-videos were feasible for GMA classification according to the Prechtl GMA standards [13] and the In-Motion instructional guideline [23]. Three (3.1%) out of 95 hospital-based videos showed inappropriate behavioral state of the infant and could not be used for GMA, leaving 92 (96.8%) of hospital-based videos feasible for GMA classification. The additional file 4 describe details about the video quality of home-and hospital-based videos.

### General movement assessment

Out of 269 scorable videos (177 taken by parents at home and 92 taken by healthcare personnel in hospital follow-up clinic), one (0.4%) video was classified with exaggerated (Fa), 12 (4.5%) with absent (FM-) and 23 (8.5%) with sporadic (FM ±) FMs. Two hundred and one (74.7%) and 32 (11.8%) videos were classified with intermittent (FM +) and continuous (FM + +) FMs, respectively.

### Parental survey

Eight families did not respond to the survey and two families did not receive the survey due to a technical error. Consequently, 85 (89.4%) out of the 95 included families responded to the survey. Forty-three (50.6%) families observed the In-Motion instructional video guide on YouTube once, 36 (42.4%) twice, and 6 (7.1%) three times. Thirty-eight (44.7%) families practiced filming their baby once before uploading and transferring a video, 33 (38.8%) twice, 6 (7.1%) three times, 3 (3.5%) four times and 5 (5.9%) five times or more.

Twenty-one percent of the respondents disagreed that the information on how GMA results would be communicated to them was good and that it was easy to keep the telephone still while filming. Seventy-eight (91.7%) respondents agreed or strongly agreed that it was easy to perform home filming. Five (5.9%) respondents agreed that they were more worried about their child`s development after filming at home. Almost 80% of respondents agreed that a video for GMA can be taken at home instead of in hospital during an out-patient visit. Details about parental responses are shown in Table 2.

**Table 2:** Details about parental responses performing home filming

	Strongly agree	Agree	Neither agree nor disagree	Disagree	Strongly disagree
It was easy to understand the information sent by SMS	70 (82.3%)	13 (15.3%)	2 (2.4%)	0	0
It was easy to find the instructional video	74 (87%)	8 (9.4%)	2 (2.4%)	0	1 (1.2%)
The information I got about when to perform filming was easy to understand	68 (80%)	16 (18.8%)	1 (1.2%)	0	0
The number of telephone calls with information about home filming was suitable	62 (72.9%)	19 (22.3%)	2 (2.4%)	2 (2.4%)	0
It was easy to do the filming without disturbing the child	39 (45.9%)	33 (38.8%)	9 (10.6%)	4 (4.7%)	0
It was easy to understand how my child should be dressed when filmed	68 (80%)	17 (20%)	0	0	0
It was easy to understand how my child should be positioned and how the mat should be when I was going to film	62 (72.9%)	23 (27.1%)	0	0	0
It was easy to follow the instructions about how the lighting should be during the filming	55 (64.7%)	20 (23.5%)	9 (10.6%)	1 (1,2%)	0
It was easy to understand how I should stand and hold the telephone during the filming	61 (71.8%)	22 (25.8%)	2 (2.4%)	0	0
It was easy to keep the telephone still while I was filming^a^	26 (31%)	40 (47.6%)	16 (19%)	2 (2.4%)	0
Filming my child for 3 min went smoothly^b^	39 (46.9%)	36 (43.4%	6 (7.3%)	2 (2.4%)	0
In general, home filming was easy to perform	57 (67.1%)	21 (24.6%)	5 (5.9%)	2 (2.4%)	0
I felt safe about uploading video of my child	48 (56.5%)	32 (37.6%	5 (5.9%)	0	0
There were no technical problems with uploading and sending the videos	34 (40%)	25 (29.4%)	10 (11.8%)	13 (15.3%)	3 (3.5%)
I became more worried about my child's development through filming at home	1 (1.2%)	4 (4.7%	22 (25.9%)	25 (29.4%)	33 (38.8%)
Performing home filming made me more attentive to my child's development	8 (9.4%)	36 (42.4%)	31 (36.5%)	7 (8.2%)	3 (3.5%)
I think parents can film at home instead of the child being filmed in hospital by healthcare personnel	27 (31.7%)	40 (47.1%)	14 (16.4%)	2 (2.4%)	2 (2.4%)
I think the information on how to get the results of the assessment was good	22 (25.9%)	25 (29.4%)	20 (23.5%)	17 (20%)	1 (1.2%)

^a^One respondent did not answer this question

^b^Two respondents did not answer this question

## Discussion

This study represents as far as we know the first knowledge translational study of remote GMA for CP risk assessment in Europe. The study shows that early filming of infant spontaneous movements both at home and at hospital visits for remote GMA can be successfully implemented in high-risk follow-up programs. Almost all parents and healthcare personnel managed to film the infants correctly and there was no substantial difference between them. Parents of high-risk infants found home-filming easy to perform, for most families it did not increase worries for their child’s development, and almost 80% agreed that filming can be done at home instead of in hospital visits. More than 50% of parents agreed or strongly agreed that filming at home made them more attentive towards their child`s development.

There are promising trends in the implementation of screening guidelines for early CP detection [20, 28–31], and two studies from the US have shown a reduction in age of CP diagnosis after implementing guidelines using a combination of GMA, MRI and HINE suggested by Novak et al. [28, 30]. A strength in our study is that it demonstrates a sensible way forward towards equal availability for early GMA screening for at-risk infants regardless of local expertise and geographical constraints. The success of performing remote GMA shown in this study may also indicate a pathway towards more efficient resource utilization. In infants with normal GMA, follow-up may be more focused on other developmental domains, whereas infants with an abnormal GMA need closer surveillance with regards to motor function. Although GMA should always be used in combination with clinical data, imaging and other standardized testing, the negative predictive value of a normal GMA is very high [5, 32].

The knowledge translation implementation framework used in this study is its core strength. Each of the three participating sites identified their individual organization’s need to implement the remote GMA as part of their follow-up program. For example, the initial information given to parents about the follow-up program, home filming and how results about the GMA evaluation were presented for parents as a part of this, were aligned and adopted according to the local hospital organization. Hence, the individualized and specific local site implementation facilitated a successful result and was supported by knowledge translational literature headlighting identification of local barriers as highly important [26]. An additional strength is the inclusion of high-risk infants typical for post-discharge NICU follow-up programs. In Norway, national guidelines recommend scheduled follow-up of high-risk infants aiming for early detection of abnormal neurodevelopment [33]. The result of this study is therefore relevant beyond the regional health authority covered by this study. With geographical restrictions and sparsely populated areas at a long distance from hospitals, remote GMA based on home-filming will facilitate equality in health care services.

Evidence of improvements in health care delivery processes using SMS communication supports our approach implementing remote GMA using an SMS based communication with parents for more widespread use of GMA as recommended in international guidelines [34, 35]. The findings in this study regarding challenges keeping the telephone still during recording and the need for improved communication on GMA results gives valuable insight for future improvements. Finally, we argue that the use of certified and experienced GMA observers for evaluation of video quality and GMA scoring, and a parental survey with high response rate, make the results trustworthy.

The current study also has several limitations. Parents in our context are, most likely, highly used to mobile health technology and there were no families that was not included in the study because they did not own a smartphone. Findings may therefore not be representative for parents/families of other ethnicities and with low employment and low education, shown to influence usage of mobile health technology [22]. Furthermore, the study aims to implement remote GMA for early CP diagnosis. Cerebral palsy outcomes are not evaluated yet, and we cannot evaluate whether there are differences in parental satisfaction between families who have a child with a CP diagnosis or not. The limited sample size also makes it difficult to rate parental responses based on the GMA classification. In parallel, our study is limited to infants with medical high-risk factors admitted to a NICU, leaving infants in primary healthcare without risk factors for CP (but with possible occurrence of CP) outside our knowledge translation approach [36]. Another limitation is the low number of included infants, making generalizability to follow-up programs with large groups of infants more uncertain.. In densely populated areas, it might be considered to translate clinical resources used for the telephone support to make implementation more feasible. However, it could be argued that our successful implementation based on a relatively small number of follow-up patients on each site will probably also make implementation feasible in high-volume settings.

Previous studies have reported on preferences of screening, assessment tools for early CP detection, and timing of diagnosis with parents of children who are diagnosed with CP [20]. More relevant and insightful for the findings in this study, would be to explore our survey findings in more detail with respect to the mixed responses of GMA result communication. Qualitative interviews will be conducted in the future with families and study site healthcare personnel to document perspectives covering more details related to these important aspects. This study presents promising results implementing remote GMA in a clinical high-risk follow-up setting. The presented solution for home-based video recordings and accompanying e-health infrastructure may also be tested for upcoming machine learning techniques for early detection of CP.

## Conclusion

This study strengthens the clinical implementation of home filming by parents to enable remote GMA for early detection of CP in high-risk follow-up programs. Consequently, it contributes to equal access to GMA independent of travel distances and local GMA competence. The implementation of remote GMA has the potential to facilitate early intervention to improve function in children with CP in line with international recommendations.

### Supplementary Information

Additional file 1. Key elements and responses of the knowledge translation action plan [27].

Additional file 2. Identified barriers and knowledge translation actions to overcome barriers.

Additional file 3. Parent survey.

Additional file 4. Compliance to Prechtl GMA standards and In-Motion instructional guides for home- and hospital-based recordings.

## Data Availability

The video data that support the findings of this study are not available due to ethical and regulatory considerations. Other data generated during the work are included in the manuscript text with accompanying tables and figures.

## Abbreviations

CP: Cerebral Palsy
GMA: General Movement Assessment
GMs: General Movements
FMs: Fidgety Movements
HINE: Hammersmith Infant Neurological Examination
NICU: Neonatal Intensive Care Unit
