# Supplementary material for: Implementation of remote general movement assessment using the in-motion instructions in a high-risk norwegian cohort
Source: BMC Pediatr. 2024 Jul 10;24:442. doi: 10.1186/s12887-024-04927-4 (PMC11234780; doi:10.1186/s12887-024-04927-4)
Supplement: Supplementary file 1 — Additional file 1. Key elements and responses of the knowledge translation action plan [27]. [file 12887_2024_4927_MOESM1_ESM.docx]

**Key elements and responses of the knowledge translation action plan (Lavis et al. 2003)**

| 1. What will we translate? | A telehealth model for remote GMA for infant risk assessment for CP in hospital-based follow-up programs |
| --- | --- |
| 1. To whom will we translate? | Practicing health professionals involved in follow-up programs in the early risk assessment for CP including pediatricians, physical therapists and nurses. |
| 1. By whom? | Chief investigator and investigator team in cooperation with local teams at St. Olavs University Hospital, Ålesund Hospital and Levanger Hospital, all in the Central Norwegian Regional Health Authority in Norway |
| 1. How will we translate? | A knowledge translational strategy to overcome clinicians recognized barriers (Supplementary Table 2). |
| 5. With what effect? | We aim to implement a model for home-and hospital-based video recordings following GMA standards, transferring video to hospital using a digital healthcare solution and establishing a remote GMA expert team for CP risk assessment. |
