# Supplementary material for: Implementation of remote general movement assessment using the in-motion instructions in a high-risk norwegian cohort
Source: BMC Pediatr. 2024 Jul 10;24:442. doi: 10.1186/s12887-024-04927-4 (PMC11234780; doi:10.1186/s12887-024-04927-4)
Supplement: Supplementary file 2 — Additional file 2. Identified barriers and knowledge translation actions to overcome barriers. [file 12887_2024_4927_MOESM2_ESM.docx]

**Identified barriers and knowledge translation actions to overcome barriers.**

| **Identified Barrier** | **Knowledge translational actions** |
| --- | --- |
| Lack of clinical time for weekly remote GMA assessments by the remote GMA team. | Prioritizing clinical activities and organization of weekly GMA remote scoring meetings by 3 experts (remote GMA team) at one remote centralized site. |
| Lack of smartphone app for hospital-based filming and sending to remote GMA scorer team. | Provide a smartphone app to enable clinicians to collect films for GMA and send the films (data) to a remote GMA team. |
| Lack of accessible instructional guides for parents filming infant movements at home. | Our research group has published evidence that it is feasible for parents to use the In-Motion instructional guides (20) through YouTube using a web-link. |
| Lack of a digital healthcare solution to make instructional guides accessible to parents and an infrastructure for transfer of home-based films with reminders complying with legislation requirements on data storage and video transfer policy. | Provide a digital healthcare solution approved for data storage and video transfer to the remote centralized GMA scorer team. The knowledge translation action will be performed in two phases: A) Use an existing digital platform sending films by an approved URL-link system and Send Short Message System (SMS) reminders manually to parents’ smartphones, B) Implement and provide a new digital healthcare solution, automatically sending SMS messages and reminders to parents, and an automatic notification to the remote GMA team when films are received. |
| Lack of parental knowledge about the early risk for CP screening performed as a part of standard follow-up programs. | Perform one telephone call to parents before every home-based filming to ensure their best knowledge and understanding of every home film procedure. |
