# Supplementary material for: Implementation of remote general movement assessment using the in-motion instructions in a high-risk norwegian cohort
Source: BMC Pediatr. 2024 Jul 10;24:442. doi: 10.1186/s12887-024-04927-4 (PMC11234780; doi:10.1186/s12887-024-04927-4)
Supplement: Supplementary file 3 — Additional file 3. Parent survey. [file 12887_2024_4927_MOESM3_ESM.docx]

**Parent survey**

Thank you for taking the time to answer these questions. It takes about 10-15 minutes to answer them. If you have questions, you can contact …, email ……, or mobile telephone …….

**1. Are you the child's mother or father?**

- Mother
- Father

**2. What is your marital status?**

- Single
- Married/cohabitant
- Separated/divorced
- Widow/widower

**3. What is your age and what is the age of your spouse/partner?**

- You:
- Your spouse/partner:

**4. How many brothers and sisters live with the child sometimes or always?**

**5. What type of mobile phone do you have?**

- iPhone (iOS/Apple)
- Android

**6. To what extent do you agree or disagree with each of these statements?**

**It was easy to understand the information sent by SMS**

- Strongly disagree
- Disagree
- Neither agree nor disagree
- Agree
- Strongly agree

**It was easy to find the instructional video**

- Strongly disagree
- Disagree
- Neither agree nor disagree
- Agree
- Strongly agree

**The information I got about when to perform filming was easy to understand**

- Strongly disagree
- Disagree
- Neither agree nor disagree
- Agree
- Strongly agree

**The number of telephone calls with information about home filming was suitable**

- Strongly disagree
- Disagree
- Neither agree nor disagree
- Agree
- Strongly agree

**It was easy to do the filming without disturbing the child**

- Strongly disagree
- Disagree
- Neither agree nor disagree
- Agree
- Strongly agree

**It was easy to understand how my child should be dressed when filmed**

- Strongly disagree
- Disagree
- Neither agree nor disagree
- Agree
- Strongly agree

**It was easy to understand how my child should be positioned and how the mat should be when I was going to film**

- Strongly disagree
- Disagree
- Neither agree nor disagree
- Agree
- Strongly agree

**It was easy to follow the instructions about how the lighting should be during the filming**

- Strongly disagree
- Disagree
- Neither agree nor disagree
- Agree
- Strongly agree

**It was easy to understand how I should stand and hold the telephone during the filming**

- Strongly disagree
- Disagree
- Neither agree nor disagree
- Agree
- Strongly agree

**It was easy to keep the telephone still while I was filming**

- Strongly disagree
- Disagree
- Neither agree nor disagree
- Agree
- Strongly agree

**Filming my child for 3 minutes went smoothly**

- Strongly disagree
- Disagree
- Neither agree nor disagree
- Agree
- Strongly agree

**In general, home filming was easy to perform**

- Strongly disagree
- Disagree
- Neither agree nor disagree
- Agree
- Strongly agree

**I felt safe about uploading video of my child**

- Strongly disagree
- Disagree
- Neither agree nor disagree
- Agree
- Strongly agree

**There were no technical problems with uploading and sending the videos**

- Strongly disagree
- Disagree
- Neither agree nor disagree
- Agree
- Strongly agree

**I became more worried about my child's development through filming at home**

- Strongly disagree
- Disagree
- Neither agree nor disagree
- Agree
- Strongly agree

**Performing home filming made me more attentive to my child's development**

- Strongly disagree
- Disagree
- Neither agree nor disagree
- Agree
- Strongly agree

**I think parents can film at home instead of the child being filmed in hospital by healthcare personnel**

- Strongly disagree
- Disagree
- Neither agree nor disagree
- Agree
- Strongly agree

**I think the information on how to get the results of the assessment was good**

- Strongly disagree
- Disagree
- Neither agree nor disagree
- Agree
- Strongly agree
