# Supplementary material for: Implementation of remote general movement assessment using the in-motion instructions in a high-risk norwegian cohort
Source: BMC Pediatr. 2024 Jul 10;24:442. doi: 10.1186/s12887-024-04927-4 (PMC11234780; doi:10.1186/s12887-024-04927-4)
Supplement: Supplementary file 4 — Additional file 4. Compliance to Prechtl GMA standards and In-Motion instructional guides for home- and hospital-based recordings. [file 12887_2024_4927_MOESM4_ESM.docx]

**Additional file 4: Compliance to Prechtl GMA standards and In-Motion instructional guides for home- and hospital-based recordings**

|  | **Home recording (n= 177), N (%)** | **Hospital recording (n=92), N (%)** |
| --- | --- | --- |
| **Standards** |  |  |
| Active movements (not hypokinetic) | 177 (100) | 92 (100) |
| Supine position | 171 (96.6) | 91 (98.9) |
| Correct state | 177 (100) | 91 (98.9) |
| No disturbances during recording | 172 (97.2) | 90 (97.8) |
| Adequate clothing | 177 (100) | 91 (98.9) |
| Whole body visible | 151 (85.3) | 85 (92.3) |
| Correct position of smartphone camera | 175 (98.9) | 88 (95.6) |
| Adequate light | 176 (99.4) | 92 (100) |
| Movements in smartphone camera potentially impacting GMA scoring |  |  |
| Optimal stability* | 152 (85.9) | 75 (81.5) |
| Abrupt displacement** | 18 (10.2) | 4 (4.3) |
| Predominantly unstable*** | 8 (4.5) | 14 (15.2) |
| Firm and comfortable base of support | 171 (96.6) | 89 (96.7) |

*****Minor/no movements in smartphone camera observed throughout the whole recording, **1-3 occasionally abrupt displacements observed in smartphone camera during recording, ***clear observable movements in smartphone camera throughout the whole recording.
